# Intercropping Enhances Arthropod Diversity and Ecological Balance in Cowpea, Hemp, and Watermelon Systems

**DOI:** 10.3390/insects16070724

**Published:** 2025-07-16

**Authors:** Ikponmwosa N. Egbon, Beatrice N. Dingha, Gilbert N. Mukoko, Louis E. Jackai

**Affiliations:** Department of Natural Resources and Environmental Design, North Carolina A&T State University, Greensboro, NC 27411, USAngmukoko@aggies.ncat.edu (G.N.M.);

**Keywords:** intercropping, pest management, crop yield, sustainable agriculture, cowpea, watermelon, industrial hemp, biodiversity, conservation

## Abstract

This study investigates arthropod assemblage in cowpea, hemp, and watermelon grown both as monocrops and intercrops using three sampling techniques: direct visual counts, sticky cards, and pan traps. A total of 31,774 arthropods were collected, spanning two classes [Arachnida (0.07%) and Insecta (99.93%)], 11 orders, and 82 families representing diverse functional groups. Arachnids were represented by a single family (Araneae). Among insects, the composition included Diptera (36.81%), Thysanoptera (24.64%), Hemiptera (19.43%), Hymenoptera (11.58%), Coleoptera (6.84%), Lepidoptera (0.076%) and Blattodea, Odonata, Orthoptera, Psocodea (≤0.005%). Roughly 10% of the total arthropods were pollinators, while the remainder were primarily herbivores and predators. Apidae were abundant in all treatments except for watermelon monocrops. Intercropping supported more pollinators, particularly Apidae, Halictidae, and Sarcophagidae. However, herbivores dominated (>50%) in each system, largely due to high presence of thrips and cicadellids. Predators accounted for approximately 30%, with dolichopodids (Diptera) being the most dominant. Watermelon yield increased by 30–60% in the intercrop systems. While intercropping increases overall arthropod abundance, it also creates a more balanced community where beneficial organisms are not heavily outnumbered by pests and contributes to enhanced ecological resilience and crop performance.

## Simple Summary

Intercropping benefits agroecosystems by modifying arthropod communities to enhance crop productivity. However, the specific roles of different crop combinations in shaping these communities remain poorly understood. This study aimed to integrate two pollinator-attractive crops (PACs) including cowpea (*Vigna unguiculata* (L.) Walp.) and hemp (*Cannabis sativa* L.) with watermelon (*Citrullus lanatus* Thunb.), a pollinator-dependent crop (PDC). We evaluated how crop combinations of a pollinator-dependent crop and pollinator-attractive crops influence pollinator and other arthropod distributions in monocrop and intercrop systems while quantifying crop yield. Intercropping supported more pollinators, particularly Apidae, Halictidae, and Sarcophagidae. However, herbivores dominated in each system, largely due to the high presence of thrips and cicadellids. Among the predators, dolichopodids (Diptera), which are important predators of aphids, were most dominant in the intercrop systems. Overall, our findings highlight the synergistic benefits of strategic crop combinations as watermelon yield increased by 30–60% in the intercrop systems compared to the monocrop. In addition, our findings indicate that although intercropping significantly increases the overall arthropod abundance, it also fosters a more balanced community where beneficial organisms are not heavily outnumbered by pests, and it contributes to enhanced ecological resilience and crop performance.

## 1. Introduction

Human-driven climate and habitat changes, particularly monocropping, are rapidly reducing insect populations. Unlike monocropping, intercropping implemented in various forms, such as mixed, row, relay, and strip planting, positively lowers the risk of species loss by offering diverse resources, enhancing ecosystem health and services, lowering farm inputs, improving soil health, and boosting crop yields [1,2,3,4,5,6,7,8]. Central to these benefits are the choice of crops, their combinations, planting patterns, the grower’s objectives, or a combination of these factors [1,2,4,6,7,8]. Some crops attract charismatic pollinators alongside predatory ones, including bees, ants, and wasps [6,7,8], which not only scout flowers for nectar and pollen but also hunt prey for food. Cropping systems designed to incorporate plants that attract pollinators, including other beneficial insects alongside crops needing pollination, can lead to increased pollinator diversity and improved harvest yields [6,7,8,9]. This is because intercropping provides diverse resources like pollen and nectar and enhances pollination services, which in turn can increase the yield of pollinator-dependent crops [6,7,8,9,10].

Intercropping systems provide valuable insights beyond pollination services and yield improvements, as well as enhanced pest management through crop diversification [3,4,6,7,8,9,11,12]. Ouyang et al. [4] showed that a rotation intercropping system involving three crops shaped predators’ preferential distribution and increased natural enemy population, which resulted in a reduction in aphid population, unlike systems with fewer crops. Similar outcomes were reported in an intercrop system with two crops [3]. In addition, intercropping pollinator-attractive crops (PACs) with a pollinator-dependent crop (PDC) enhanced insect diversity and increased the yield of a pollinator-dependent crop [6,7,8]. Knowledge of the specific crop choices and the combinations may help redress some monocrop-associated pest challenges [1,2,3,4,6,7,8,9]. The response of aphids to predators in a system with the right crop combinations [3,4] lends credence to the value of crop choices in intercrop systems. The choices and/or combination of pollinator-dependent crops (PDCs) and pollinator-attractive crops (PACs) lead to improved crop yield [8].

Intercropping also improves pollinators and predators’ activities [3,4,6,7,8]; however, much information is still lacking on the role of intercropping on the arthropod community. This study aims to incorporate pollinator-attractive crops (PACs) such as cowpea (*Vigna unguiculata* (L.) Walp.) and hemp (*Cannabis sativa* L.) with pollinator-dependent crops (PDCs), including watermelon (*Citrullus lanatus* Thunb.), to evaluate how crop combinations influence the distribution of pollinators and other arthropods in two cropping systems (monocrop and intercrop) while quantifying crop yield. We hypothesize that intercropping with the right crop combination will lead to increased pollinator numbers, diversity, and crop productivity. This would offer unique insights into understanding the arthropod community, the agricultural ecosystems, and their management. This research helps understand how some farming practices influence the distribution and abundance of beneficial and pest arthropods, which are crucial for maintaining ecosystem health and crop yields.

## 2. Materials and Methods

### 2.1. Study Site and Plants

The study was conducted in summer 2023 at the North Carolina Agricultural and Technical State University Research Farm (36.0613° N, 79.7361° W) in Greensboro, NC, USA. Watermelon (*Citrullus lanatus),* a pollinator-dependent crop (PDC), purchased from Johnny’s Selected Seeds (Winslow, ME, USA), and two pollinator-attractive crops (PACs), industrial hemp *Cannabis sativa* L. (Joey variety) from King’s Agriseeds, Lancaster, PA and cowpea *Vigna unguiculata* (Pink-eyed Purple Hull variety) from Johnny’s Selected Seeds (Winslow, ME, USA), were used in this study. The selections were driven by the high preference for hemp pollen and cowpea nectar by pollinators as pollinator-attractive crops [13,14] and the dependency of watermelon on pollinators. In addition, watermelon is a crop grown and consumed widely in the USA.

### 2.2. Experimental Design

In the monocrop system, the crops were grown individually (Cowpea, Hemp, and Watermelon) as control, and the intercrop system consisted of two crops planted in strips (Hemp + Cowpea, Watermelon + Cowpea, and Hemp + Watermelon). The intercrops were laid out in a randomized complete block design (RCBD) with four replications, and the monocrops were planted in a separate plot about 10 m away from the intercrop treatments and separated by 2 m wide and 15 m long corn rows. Each intercrop treatment comprised two rows per crop for a total of four rows per treatment. The rows were 5 m long with 1 m inter-row spacing within the treatments and replicated four times with 2 m spacing between blocks. The control plots consisted of two rows, 5 m long and 1 m between rows, and were replicated three times. The planting dates were staggered for the different crops to synchronize their flowering. Cowpea was planted on 31st May 2023, Hemp on 6th June 2023, Watermelon on 9th June 2023, on a field mulched with wheat straw obtained from Rankin Farms Inc., Ellerbe, NC, USA. Standard practices for growing organic vegetables were followed, with no pesticide applications. All plots were irrigated with sprinklers as needed weekly using locally sourced water from a nearby pond.

### 2.3. Sampling

The arthropods associated with each treatment at flowering were examined using three sampling techniques (direct visual counts, pan-trap, and sticky cards) for five weeks. With direct visual observations, a predetermined group of pollinators (honeybees, bumble bees, carpenter bees, sweat bees, and wasps) was counted on each 5 m row for 60 s. The visual count of pollinators commenced on 17th July 2023 (at the beginning of flowering) and continued weekly for five weeks (14 August 2023) between 08:00 and 10:00 (when pollinators are generally active and also easy for the counts to be conducted).

Sampling with blue, white, and yellow pan traps was carried out once weekly from 19th July 2023, following the presence of flower visitors, and concluded on 16th August 2023. As described by Dingha et al. [6,7], the traps were made from 16 oz squat polypropylene deli bowls (BioServ, Frenchtown, NJ, USA) painted yellow for the yellow trap, UV-bright fluorescent blue for the blue trap, and white for the white trap. Unpainted bowls were used as pan-trap holders by gluing each onto a metallic prop with a circular base at the top. The props and affixed bowls were mounted in the middle rows within treatments. The painted trap bowls were labelled, mounted on the prop, and an unscented soapy water solution was added up to about half their capacity and left in the field. After 24 h, the trap bowls were strained off the soapy water through their lids fastened with fine mesh to keep insects enclosed while draining and then taken to the laboratory for further processing (specimen cleaning, preservation in 70% alcohol, and identification). All traps were collected in the order of deployment to ensure approximately equal exposure time.

Sampling was also conducted using double-sided yellow sticky cards (8 cm × 13 cm) (Sticky Strips, Olson Products, Medina, OH, USA). The technique facilitates capturing smaller-bodied pollinators that escaped sampling by visual counts and pan traps [7]. Weekly sampling with sticky cards began on 18th July 2023 and was terminated on 15th August 2023. The sticky traps were attached to metal stakes positioned in the middle of each inner row of each treatment, at the same height as the crops. The traps were retrieved after 24 h and placed in Ziploc^®^ bags for further processing using a stereomicroscope (Olympus SZX7, Japan).

Direct visual count of pollinators was identified by pollinator types (honeybees, bumble bees, carpenter bees, sweat bees, and wasps). Pollinators trapped on sticky cards were identified to the family level due to difficulty in specimen maneuverability on the cards. Those from pan traps were identified to the species level. Therefore, visually sampled insects were identified by their common names, while those sampled with pan traps and sticky cards were identified using dichotomous keys [15,16] and other freely accessible online resources, namely Bug Guide (https://bugguide.net/), GBIF (www.gbif.org), and Discover Life (https://www.discoverlife.org/), accessed continually as needed from 10 October 2023 through to 30 July 2024.

### 2.4. Crop Yield

Crops in each treatment, including the control, were harvested at maturity, except for hemp, whose seeds were depredated by granivorous birds. At maturity (from ca. 83–127 days), watermelon fruits were counted and weighed, and twenty-five dry pods from each cowpea treatment (~106 days after planting) were randomly harvested and weighed. Harvested fruits and pods were weighed using an Ohaus T51P weighing scale (Pine Brook, NJ, USA), and data were recorded.

### 2.5. Data Analyses

Total pollinator counts from direct visual observation, pan traps, and sticky cards were separately analyzed for diversity indices [total individuals, taxa richness, dominance (D), Shannon (H’), evenness (E), and Margalef indices] using PAST™ version 4.03 [17]. Shannon and dominance were converted to first- and second-order Hill numbers [18]. While total individuals represent the sum of all pollinators captured within a cropping system, the taxa richness represents the total number of unique taxonomic categories (or pollinator type in visual counts) within a cropping system (or treatment). Dominance ranges from 0 (with equal presences of all taxa) to 1, where one taxon completely dominates the community [17]. Shannon diversity index utilizes the number of individuals and the number of taxa to model community diversity. Communities with numerous taxa and few members within each taxon are highly diverse, while a community with a single taxon is least diverse and has zero H’ index, while evenness measures taxa equitable distribution and ranges from 0 to 1 [17,19,20]. Total pollinator counts for visual observations were analyzed with a zero-inflated negative binomial regression owing to the presence of excess zeros; for pan traps, Kruskal–Wallis was employed using ‘agricolae’ package version 1.3-7 [21], and for sticky cards, a generalized linear mixed model with blocks treated as random effects was utilized using ‘glmmTMB’ package version 1.1.10 [22]. Total counts of herbivores and predators were analyzed using ANOVA, as they satisfy the assumptions of normality and homogeneity of variance. In the advent of significant differences in global analyses, appropriate post hoc tests were adopted using ‘emmeans’ version 1.10.4 [23] and ‘multcomp’ version 1.4-26 [24] packages with Tukey or Sidak adjusted *p*-values to distinguish the statistically different means (alpha set at 5%). Pearson’s product moment correlation was used to correlate herbivores and predators’ total counts. Cowpea and watermelon yields were analyzed using a generalized linear mixed model, with cropping systems and blocks considered as fixed and random effects, respectively (alpha = 5%), and means separated as stated previously. All descriptive statistics were summarized using the ‘FSA’ package version 0.9.5 [25], and all analyses were conducted in R 4.4.1 [26]. Detrended Correspondence Analysis (DCA) was used to explore arthropod taxa composition across cropping systems, treated as categorical variables, and taxa positioned with non-overlapping text labels [27].

## 3. Results

### 3.1. Pollinator Abundance and Diversity in Intercrop and Monocrop Systems

Visual observation accounted for 735 individual pollinators, spanning five types (honeybees, bumble bees, carpenter bees, sweat bees, and wasps) across three families, with the highest number (228) on Watermelon + Cowpea intercrop and the lowest (24) on Hemp monocrop (Table 1). There were more honey bees (χ^2^ = 13.58, *p* = 0.019), bumble bees (χ^2^ = 61.85, *p* < 0.001), carpenter bees (χ^2^= 14.73, *p* = 0.011), and wasps (χ^2^ = 16.04, *p* = 0.007) in the intercrop treatments than the monocrops, but sweat bees were evenly distributed (χ^2^ = 9.14, *p* = 0.104) (Table 1). All the pollinator types were recorded in all the intercrop treatments; however, they varied between three and four in the monocrop treatments. Pollinator dominance (D) was lowest in the Watermelon + Cowpea intercrop and highest in the Watermelon monocrop treatment. Shannon index showed the Watermelon + Cowpea intercrop had the most diverse pollinators, while Watermelon monocrop treatment was the least diverse (Table 1).

Pan traps captured 389 individual pollinators across 51 species in 14 families, from which Halictids were the most abundant, while Syrphidae, Cantharidae, Andrenidae, Scoliidae, and Sphecidae occurred as singletons (Table 2). The Watermelon + Cowpea intercrop treatment had the most species (29) and individual count, unlike Hemp, which had the least (Table 2). Whilst dominance was comparatively similar and generally low across all treatments, the Shannon index ranged from 2.03 to 2.67, and the Margalef index ranged from 2.25 in Hemp to 6.14 in Watermelon + Cowpea intercrop (Table 2). The pollinators caught in pan traps had low dominance, which ranged from 0.11 to 0.15. Hemp monocrop treatment had the highest species evenness compared to the other treatments (Table 2).

Sticky cards recorded 2229 individual pollinators from 16 families, mostly dominated by Sarcophagidae and Halictidae, with the least representation from the lepidopteran families (Table 3). Sarcophagidae were most abundant in the Cowpea + Hemp intercrop treatment, followed by Watermelon + Cowpea and Cowpea monocrop treatments, but comparatively low in the Hemp monocrop treatment. Halictidae was lowest in the Hemp and Watermelon monocrop treatments and fairly even across the intercrop treatments, with Watermelon + Cowpea and Hemp + Watermelon recording the highest numbers (Table 3). Taxa count ranged from 8 to 13, with Watermelon + Cowpea intercrop recording the highest total individuals (608), unlike the Hemp (103) monocrop. Except for Cowpea, the monocrops had the least pollinator diversity (Shannon diversity indices), and dominance indices were homogenously low regardless of treatments (Table 3).

### 3.2. Total Pollinators

**Figure 1:**
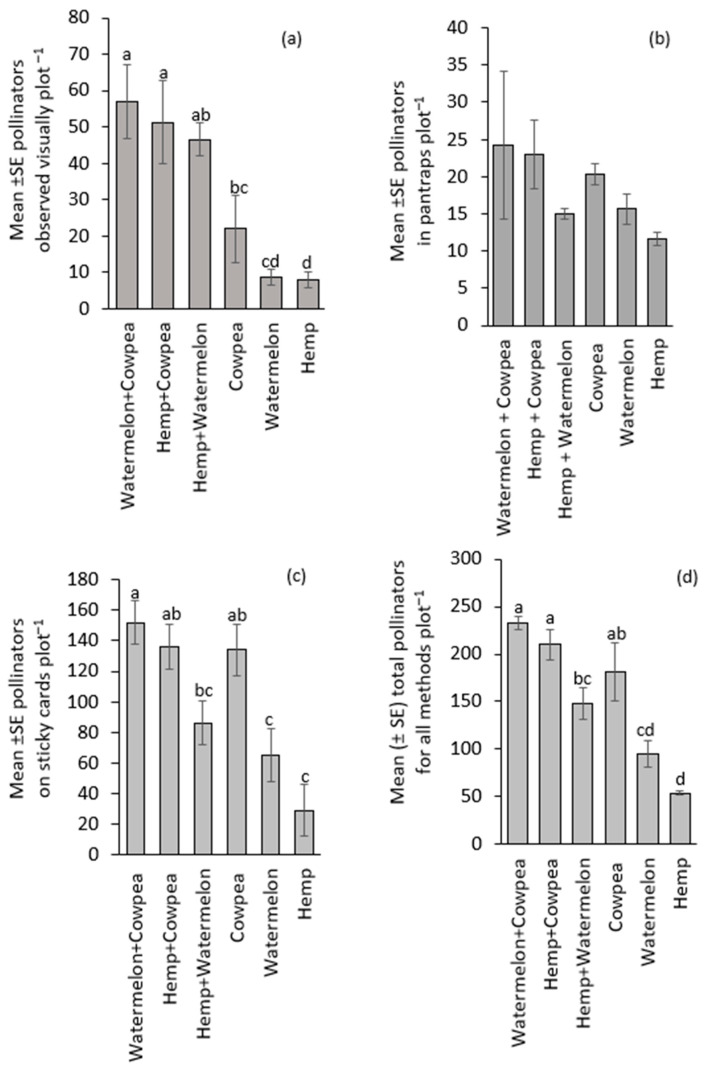
Pollinator abundance observed visually through (**a**) direct visual counts, (**b**) pan traps, (**c**) sticky cards, and (**d**) all three sampling methods combined. Treatment means (bars) within each graph followed by the same letter(s) are not significantly different (Sidak-adjusted *p*-value to correct for family-wise error, *p* > 0.05). The absence of letters above the bars indicates that no statistically significant differences were found among the treatments (*p* > 0.05).

Visual counts revealed a significant uneven distribution of pollinators among the cropping systems (F_(5,15)_ = 11.36, *p* < 0.001). Each of the intercrop treatments recorded higher pollinator numbers than their corresponding monocrop treatments (Figure 1a). However, in pan traps, Watermelon + Cowpea, Hemp + Cowpea intercrops, and Cowpea monocrop had high number of pollinators, which did not differ significantly from the other treatments (H = 5.6, df = 5, *p* = 0.33; Figure 1b). Sticky cards caught more pollinators in the Watermelon + Cowpea, intercrop treatment than in the Watermelon monocrop treatment (Wald χ^2^ = 57.43, *p* < 0.001; Figure 1c). Combining all three sampling methods, the number of pollinators recorded in the intercrop systems (Watermelon + Cowpea and Hemp + Cowpea) and the Cowpea monocrop was significantly greater than that in the Hemp and Watermelon monocrop treatments (Wald χ^2^ = 121.64, *p* < 0.001; Figure 1d).

### 3.3. Total Predator and Herbivore Distributions

**Figure 2:**
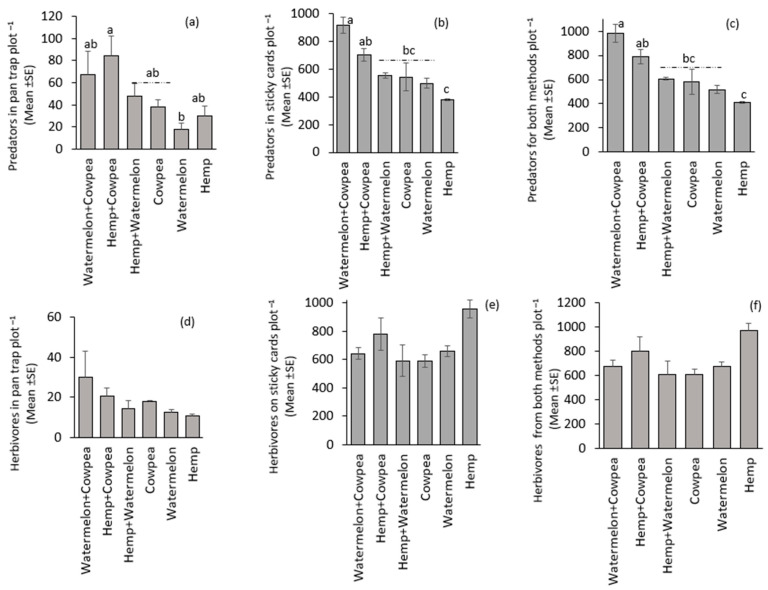
Abundance of predators in (**a**) pan traps, (**b**) sticky cards, and (**c**) both pan traps and sticky cards, and herbivores in (**d**) pan traps, (**e**) sticky cards, and (**f**) both pan traps and sticky cards. Treatment means (bars) within each graph followed by the same letter(s) are not significantly different (Sidak-adjusted *p*-value to correct for family-wise error, *p* > 0.05). The absence of letters above the bars indicates that no statistically significant differences were found among the treatments (*p* > 0.05).

**Figure 3:**
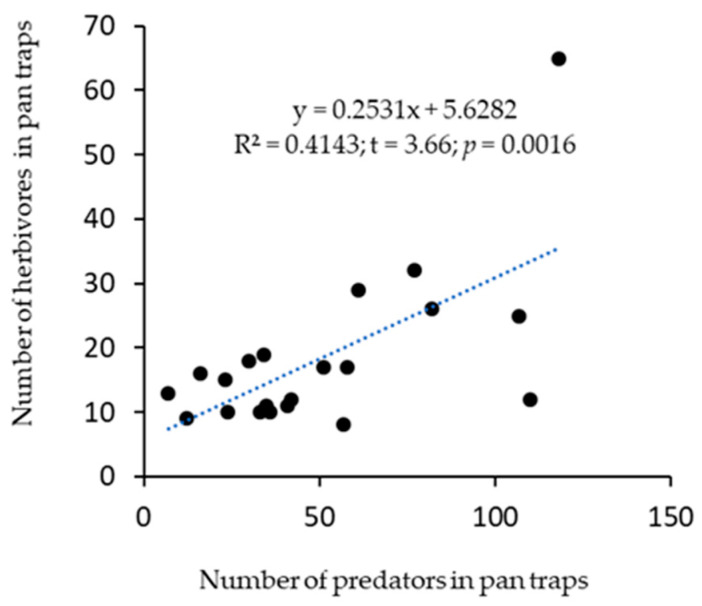
Correlation between the number of predators and herbivores trapped in pan traps.

The cropping systems influenced the distribution of predators. For example, from the pan trap, significantly (F(_5,15)_ = 3.06, *p* = 0.042) fewer predators were recorded in the Watermelon monocrop, and the highest predators were in the Hemp + Cowpea intercrop treatment (Figure 2a). Similarly, predators caught in sticky cards were significantly (F(_5,15)_ = 3.06, *p* = 0.042) different, with the highest catch in Watermelon + Cowpea intercrop (Figure 2b). When both pan traps and sticky cards catches were combined, predator distribution among treatments was significantly different (F(_5,15)_ = 12.16, *p* < 0.001) (Figure 2c). There was no significant difference among treatments on the distribution of herbivores in pan traps (F(_5,15)_ = 1.22, *p* = 0.35), and sticky cards (F(_5,15)_ = 2.64, *p* = 0.07). Nonetheless, the Hemp monocrop had the lowest value in pan traps and the highest in sticky cards (Figure 2d,e). In combined catches from both pan traps and sticky cards, herbivore distribution among the treatments was not significantly different (F(_5,15)_ = 2.42, *p* = 0.08) (Figure 2f). However, there was a positive and significant relationship (r = 0.644; t = 3.66; *p* = 0.0016) between herbivore incidence and the number of predators captured in pan traps (Figure 3). No relationship (r = −0.165; t = −0.729; *p* = 0.475) was observed between herbivore and predator from sticky cards and combined pan-trap and sticky cards catches (r = −0.066; t = −0.29; *p* = 0.775).

### 3.4. Total Arthropods

Thirty-one thousand, seven hundred and seventy-four (31,774) individuals belonging to 82 families, 11 orders, and 2 classes (Arachnida and Insecta) of arthropods were recorded from the six cropping systems involving monocrops (Cowpea, Hemp, and Watermelon) and intercrops (Hemp + Cowpea, Hemp + Watermelon, and Watermelon + Cowpea) in this study (Table 4). The most abundant order was diptera (36.8%), with 12 families from which Dolichopodidae was most prevalent (50.33%) and highest (28.05%) in the Watermelon + Cowpea intercrop and least abundant (7.61%) in Cowpea monocrop treatment (Table 4). The second most abundant dipteran Chloropidae (25.23%) was mostly recorded in the Watermelon + Cowpea intercrop (33.28%) and was least present in the Watermelon monocrop treatment (5.96%). Thysanoptera (24.64%) was the second most abundant insect order and was solely represented by the family Thripidae, which was most abundant in the Hemp monocrop (26%), followed by Hemp + Cowpea (24%), and least accounted for in the Cowpea monocrop (7.7%).

Next in order ranking was the order Hemiptera (19.43%), represented by 21 families, including Cicadellidae (56.82%) and Aphididae (19.35%), which are economically important pests. The Cowpea monocrop treatment accounted for 21.87% of the cicadellids observed, followed by Hemp + Cowpea (21.1%) and Watermelon + Cowpea (21.1%), while Hemp + Watermelon (10.5%) had the smallest count. Aphid counts were highest in Hemp + Watermelon (27.89%) and Watermelon + Cowpea (19.6%) and lowest in Cowpea monocrop (7.87%). The mirids were highly abundant in the Hemp monocrop (27.25%) and the Hemp + Cowpea intercrop (24.6%) and least abundant in the Cowpea monocrop (8.73%). The anthocorids, known for their predatory prowess, were 5.59% of the total hemipterans. They were most numerous in the Watermelon + Cowpea intercrop (26.4%) and the Hemp Cowpea (21.1%) intercrop and lowest in the Hemp monocrop (3.77%).

The fourth ranked insect order was Hymenoptera, representing 11.58% of all arthropods, but it was the most prevalent taxon with 22 families. Of these families, the predatory Scelionidae (26.8%) and the flower-visiting Halictidae (20.9%), Braconidae (16.4%), and Apidae (13.7%) were the most plentiful. The Scelionidae were fairly distributed across the intercrops (Watermelon + Cowpea [27.4%], Hemp + Cowpea [23.9%], and Hemp + Watermelon [18.5%], which compares closely to the Cowpea monocrop (17.1%), and least in Hemp (5.1%). Similarly, the halictids explored the intercrops on an average of 20%, trailed closely in the Cowpea monocrop (15.2%), but they were least abundant in Hemp (8.2%). Numerous other families, including Cynipidae, Pompilidae, and Vespidae were also present (Table 4).

Coleoptera is ranked as the fifth most abundant insect order, with 13 families dominated by the herbivorous Chrysomelidae (45.5%) and predatory Coccinellidae (22.97%). Approximately one-third (27.23%) of the chrysomelids were reported in the Watermelon monocrop treatment, Hemp + Watermelon (22.37%), Watermelon + Cowpea (19.23%), and Hemp + Cowpea intercrops (13.77%) (Table 4).

Functionally grouping arthropods revealed herbivores to be the most prominent group, with about 55 to 76% occurrence, highest in the Hemp monocrop (75.9%) and lowest in the Watermelon + Cowpea intercrop. These were followed by predators and pollinators; meanwhile, some taxa exhibited overlapping functional traits, such as three in Cantharidae (coleoptera), Ceratopogonidae (Diptera), and Formicidae (Hymenoptera), two in Elateridae, Mordellidae, Scarabaeidae (Coleoptera), even though there are also taxa like Anthocoridae (Hemiptera) with only a single functional group (Table 4). Cowpea had the most diverse community of arthropods (Shannon index = 2.793; Hill number of order one (^1^*D*) = 16.3) and the Hemp monocrop the least diverse (Shannon index = 2.059; Hill number of order one (^1^*D*) = 7.8). The Hill numbers of order two (^2^*D*), which reflect the effective number of dominant species, show that some assemblages were more evenly distributed (e.g., ^2^*D* = 10.0), while others (e.g., ^2^*D* = 4.0) were more heavily dominated by a few families. All the other four cropping systems (Watermelon monocrop and the three intercrops) were numerically similar for both indices. A > 5.0, Margalef indices showed exceptionally high species richness across all six cropping systems, which in turn had low dominance (Table 4).

**Figure 4:**
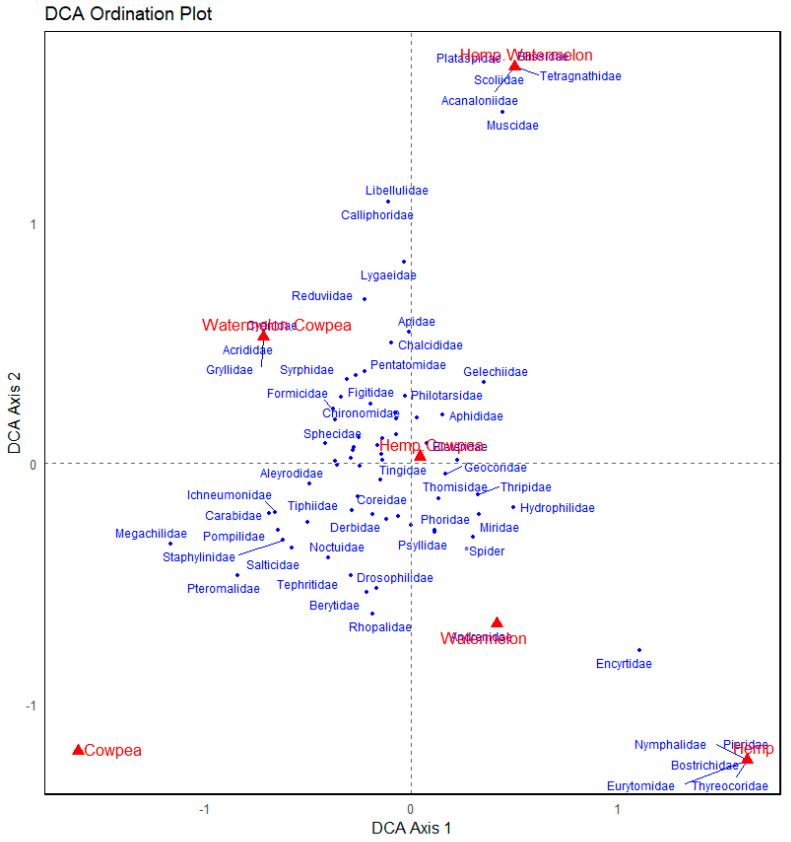
Detrended Correspondence Analysis (DCA) ordination of arthropod communities across different cropping systems. Each point represents an arthropod family, with proximity in ordination space reflecting similarity in community composition. The cropping systems are in red prints, while arthropod families are in blue. Seventeen (17) data points were unlabeled due to overlaps.

The Detrended Correspondence Analysis (DCA) plot shows some distinctions in arthropod community composition across cropping systems. The Cowpea monocrop was strongly separated along the primary axis (DCA axis 1; Figure 4) and associated with pollinators and beneficial taxa such as Megachilidae, Pompilidae, and Carabidae, indicating a unique and specialized community. Meanwhile, Hemp and its combinations (Hemp + Cowpea and Hemp + Watermelon) cluster on the opposite side, linking families like Nymphalidae, Pieridae, and Thyreocoridae, suggesting a different ecological assemblage, possibly more herbivorous or pest-related. Watermelon appears ecologically distinct as well, positioned far along both axes, and associated with a narrower set of taxa like Andrenidae. The intercropping cropping systems (e.g., Watermelon + Cowpea) showed broader associations with diverse arthropod families, indicating more generalized communities or overlapping niches, that is, close positioning of multiple families in the ordination space, indicating frequent co-occurrence across cropping systems and likely reliance on similar resources. This spatial proximity suggests the shared ecological roles or functional redundancy. That many arthropod families clustered around the intercropping systems implies their suitability for a broad range of communities, with potential for resource sharing, coexistence, or competition. In sum, the ordination reveals that cropping combination sharped the composition of arthropod communities, with Cowpea attracting pollinator-rich assemblages, Hemp supporting a distinct suite of species, and intercrops fostering broader biodiversity.

### 3.5. Crop Yield Among Treatments

**Figure 5:**
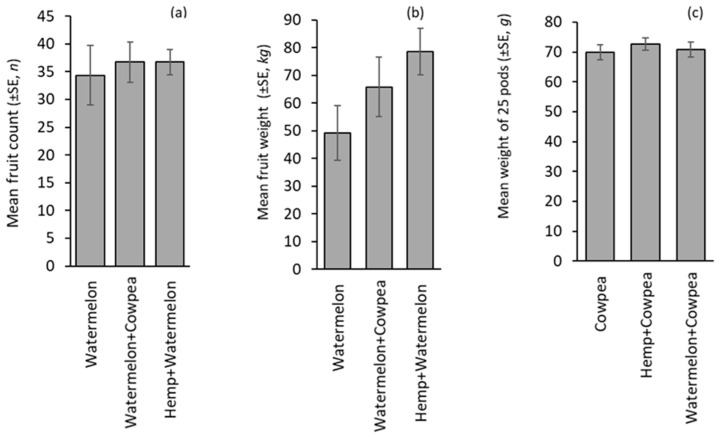
Crop yield: (**a**) watermelon fruit count, (**b**) watermelon fruit weight, and (**c**) cowpea dry pod weight in monocrop and intercrop. The absence of letters above the bars indicates that no statistically significant differences were found among the treatments (*p* > 0.05).

The fruit counts for watermelon did not differ significantly by treatments (F_(2,8)_ = 0.13, *p* = 0.879) but were numerically higher in the intercrop than the monocrop (Figure 5a). Additionally, the weight of watermelon fruits was 34% and 60% higher in Watermelon + Cowpea and Watermelon + Hemp intercrops than the Watermelon monocrop, which had 49.20 ±9.92 kg, although it was statistically similar (F_(2,8)_ = 2.099, *p* = 0.185) (Figure 5b). Cowpea yield in monocrop and intercrop systems was statistically similar (F_(2,8)_ = 0.324, *p* = 0.727; Figure 5c). Seed weight from hemp was not recorded as seeds were destroyed by birds before harvest.

## 4. Discussion

Cropping systems are inseparably tied to numerous beneficial, problematic, and neutral arthropods [8,28,29,30,31]. In this study, the composition and abundance of arthropods associated with six cropping systems (three monocrops and three intercrops), using three sampling methods, revealed that about a tenth of the total arthropods observed (31,774) were pollinators. Additionally, the Watermelon + Cowpea (i.e., pairing a pollinator-dependent crop with a pollinator-attractive crop) and Hemp + Cowpea (two pollinator-attractive crops) recorded the most pollinators and the Hemp monocrop recorded the least (Figure 1d). This contrasts with a single-crop system, where over 80% of the reported insects on hemp were pollinators [32]. Yet another study reported eight insect orders and two non-insect mollusks on hemp [31], whereas in this study, six insect orders and one non-insect order (Araneae) were reported. Several factors could have contributed to these differences, including the sampling method used [6], the hemp variety [13], the cultivation practices, indoor or outdoor conditions [31], and the aim of the study. From our findings (Table 1), Apidae, mainly bumble bees, were the most prominent pollinators in direct visual count, particularly in the intercropping systems, probably because of their large size and easy recognition [7,13]. The abundance of these bees, especially in Hemp + Watermelon and Watermelon + Cowpea intercrops, has not been previously reported, but it partly aligns with the taxon’s prominence documented in Hemp + Squash and Squash + Cowpea intercrops [8]. This outcome is unsurprising because squash (*Cucurbita* sp.) and watermelon [*Citrullus lanatus* (Thunb.) both are pollinator-dependent crops (PDCs) and belong to the same Cucurbitaceae family.

Intercropping contributed to a greater presence of bees foraging on the crops compared to monocrops (Figure 1). This aligns with reports from other studies of low pollinator numbers on monocrops. The authors attributed this to climatic constraints and limited foraging options [33,34]. Nonetheless, high honey bee numbers have been reported on crops such as hemp, watermelon, and cowpea in a monocropping system [6,7,13,32,34].

Other plausible reasons for the greater bee counts in intercropping systems could be the interplay of floral resources, such as copious pollen and nectar from pollinator-attractive crops like hemp and cowpea. Intuitively, when carbohydrate-rich nectar and protein-rich pollen are more readily available in an intercropping system, they may reduce foraging efforts by providing easy access to abundant food. This is likely to attract more pollinators than isolated foods, best described as ‘situational awareness’. Pollinators’ capacity to perceive nutritious food sources and the presence of competitors (predators) in their environment has been observed in bees, including honey bees, which actively avoid risky food sources, due to the presence of a Crab spider *Xysticus elegans* (Araneae: Thomisidae), an euryphagous predator [35,36,37]. Understanding these dynamics and behaviors (situational awareness) can help shape how crop pairings for polyculture are framed to boost crop productivity and hence how the arthropod community shapes the behavior of other fauna.

In the current study, visual observation revealed twice as many pollinators as those captured in pan traps. However, identifying insects at the species level through visual observation is challenging. This limitation was overcome with pan trap counts, where halictid bees represented four genera and several species, including *Agapostemon* species, *Augochlora pura*, *Halictus* species, and *Lasioglossum viridatum*. These bees were the most abundant pollinators, especially in the intercropping systems compared to monocrops (Table 2). In [38], *Lasioglossum* species spent more time on watermelon flowers than other pollinators, visiting a flower four times for a successful fruit set, even though a single pollen transfer can suffice. In contrast, at least eight visits from a honey bee are needed to achieve the same outcome [39]. The role of *Lasioglossum* in pollination highlights the importance of native bees in crop production. Such roles reinforce the need to protect, conserve, and sustainably support wild bee populations as an integral component of agricultural strategies and/or systems. Additionally, Apidae, represented by six genera and seven species, including *Apis mellifera*, *Bombus impatiens*, *B. pensylvanicus*, and *Ceratina strenua* were recorded in the pan trap. Conversely, sticky cards attracted diverse insects, including Sarcophagidae (Table 3). Some sarcophagid flies are predators of Halictidae bees [40], and this behavior can influence pollinator abundance and foraging behavior. Our findings reveal a greater abundance of sarcophagid flies in the treatments that recorded high numbers of Halictidae (Table 1 and Table 2). Furthermore, we identified three predatory spiders (Salticidae, Tetragnathidae, Thomisidae) (Arachnida: Araneae), which hunt both harmful and beneficial insects as well as plant pollen [29,35,36,41,42]. The most abundant wandering spiders (salticids and thomisids) can consume approximately 34 species of insects from 21 families and 9 orders and outperform web spinners [41]. These examples indicate that the foraging behavior of pollinators can be influenced by the struggle between maximizing food intake and minimizing predation risk.

Among the thirteen coleopteran families, Chrysomelidae include important herbivorous flea and spotted cucumber beetles that can defoliate crops and reduce crop productivity. Several examples of hemp-associated chrysomelids include *Altica* sp., *Colaspis* sp., *Phyllotreta* species, *Diabrotica balteata* LeConte, and *D. undecimpunctata howardi* Barber [29,30]. Chrysomelids were notably present across cropping systems in this study, suggesting crops face a fairly even risk of herbivory from these pests. It is worth mentioning the incidental presence of termites (Blattodea) in the Hemp + Cowpea intercrop. However, *Reticulitermes* sp. and *Coptotermes formosanus* found on *Cannabis sativa* were linked to wood mulching [30], an assumption that parallels the straw mulching used for land preparation in this study. These pests also had predators such as coccinellids (lady beetles), dolichopodids (long-legged flies), carabids, and others (Figure 3). Dolichopodids are important predators of aphids, psyllids, fungus gnats, chironomids, leafhoppers, thrips, whiteflies, and hymenopterous parasitoids [43,44]. Nevertheless, carabids were much more prevalent in cowpea-associated treatments than in the others. Rove beetles (Staphylinidae) were few overall and absent in the watermelon monocrop. While not categorized as pests or beneficial on hemp, they are important flower visitors known for aiding pollination [29]. Predator–herbivore associations often influence each other, and the substantial presence of predator and prey (Figure 3) suggests the key predatory role of dolichopodids in this study, perhaps driven by the rich native flora surrounding the study site [45]. These examples highlight the functional diversity of coleopterans (including other orders) in agroecosystems, which extends beyond pestilence to provide beneficial services as predators and pollinators [29,30,46,47].

The contribution of predatory arthropods to pest-control services is valued at an estimated USD 4.49 billion annually [48]. For example, the availability of multiple-host plants (aphid-rich diets) influenced the distribution of predatory coccinellids (*Coccinella algerica* and *Hippodamia variegata*) [49]. Another study involving a three-crop intercrop complex indicated that intercropping promoted greater aphid suppression by natural enemies compared to monocrops [4]. These findings align with our results, where cropping systems shaped predator choices (Figure 2a–c; Figure 3). This emphasizes how intercropping sustainably fosters resilient and robust farming systems, which mitigates monocrops’ effects on ecosystem processes, composition, structure, flora–fauna and fauna–fauna interactions in favor of biodiversity [8,10,50]. The United States’ policy on sustainable agriculture promotes the protection and preservation of all species and biological interactions while simultaneously advocating for nature-based solutions to sustainability challenges [50]. Consequently, all arthropods are essential biological resources whose ecological roles significantly contribute to human well-being and sustainability.

From our findings, watermelon recorded higher fruit count and weight with a 34–64% yield increase in the intercrop systems compared to the monocrop. The presence of floral resources (pollen and nectar) at the right time in a cropping system to attract diverse and abundant pollinators and predators could have contributed to the observed increase in yield of the pollinator-dependent crop, as reported in other studies [7,8,14]. In addition, crop types, pairings, planting dates, and spacing are central attributes to maximizing yield with minimal inputs [51,52,53]. Incorporating legumes and a pollinator attractive crop like cowpea with their symbiotic nitrogen-fixing capabilities enhances soil nitrogen [51] amongst other benefits. It is also noteworthy that in some instances, monocropping can outperform intercropping under certain conditions, particularly when reduced competition for nutrients leads to more available resources [52,54,55]. These examples emphasize the importance of context-specific evaluations of intercropping systems in sustainable pest management. Such assessments should be aimed at enhancing yield through increased pollination and reduced pest pressures [3,4,56,57]. Considering the perspectives of Blackwell et al. [58], the yield loss of hemp to granivore’s invasion highlights avian pest risk to profitable hemp seed production. This was the experience in our study, and it brings to light the value of hemp seeds for the commercial production of bird feeds, especially for domesticated birds, a concept worth exploring further.

## 5. Conclusions

Understanding the impact of intensive agriculture on arthropod presence, distribution, abundance, and functional roles is essential for sustainable crop production while minimizing effects on native biodiversity. However, adopting intercropping with flowering crops requires careful consideration of phenological nuances, as the timing and counts of flower initiation may vary among different varieties. If not carefully synchronized, this may lead to desynchronized blooming across crops. Such misalignment can disrupt vital arthropod–floral interactions and undermine the ecosystem benefits thereof. Synchronizing pollination-dependent crops (PDCs), which rely on floral visitors for productivity, with pollinator-attractive crops, as seen in this current study, not only enhances crop yields but also helps control herbivore populations through predatory regulation. Supporting this with our findings, watermelon (a pollinator-dependent crop) exhibited the lowest pollinator counts and yield when planted as a monocrop compared to watermelon intercropped with either hemp or cowpea, which are both attractive to pollinators. Although the attraction of pollinators, whether in monocrop or intercrop, does not significantly affect its productivity due to its ability to self-pollinate, its role enhances the companion intercrop by attracting pollinators and supplementing nutrients. This specifically highlights the synergistic benefits of strategic crop combinations.

## Figures and Tables

**Table 1:** The average number of pollinators and the total counted using direct visual counts on the monocrop and intercrop treatments.

Family	Pollinator Types (Common Names)		Mean Pollinator Count ± SE (Total Count) *					**Statistics**
		Cowpea	Hemp	Watermelon	Hemp + Cowpea	Hemp + Watermelon	**Watermelon + Cowpea**	
Apidae	Honey bees	3.67 ± 1.20 ^ab^ (11)	4.33 ± 0.88 ^ab^ (13)	0.33 ± 0.33 ^b^ (1)	4.50 ± 2.63 ^ab^ (18)	8.00 ± 1.47 ^a^ (32)	7.50 ± 1.44 ^a^ (30)	χ^2^ = 13.58, *p* = 0.019
	Bumble bees	2.67 ± 0.33 ^b^ (8)	2.67 ± 0.88 ^b^ (8)	6.00 ± 2.65 ^b^ (18)	21.50 ± 5.20 ^a^ (86)	31.0 ± 3.72 ^a^ (124)	26.25 ± 3.40 ^a^ (105)	χ^2^ = 61.85, *p* < 0.001
	Carpenter bees	0.00 ± 0.00 ^b^ (0)	0.00 ± 0.00 ^b^ (0)	0.00 ± 0.00 ^b^ (0)	2.25 ± 0.25 ^a^ (9)	0.75 ± 0.25 ^ab^ (3)	1.75 ± 0.85 ^ab^ (7)	*H* = 14.73, *p* = 0.012
Halictidae	Sweat bees	3.67 ± 2.3 (11)	0.00 ± 0.00 (0)	2.33 ± 1.33 (7)	2.00 ± 1.08 (8)	4.75 ± 1.11 (19)	2.94 ± 1.47 (20)	*H* = 9.14, *p* = 0.104
Vespidae	Wasps	12.00 ± 5.50 ^ab^ (36)	1.00 ± 0.58 ^ab^ (3)	0.00 ± 0.00 ^b^ (0)	21.00 ± 5.9 ^a^ (84)	2.00 ± 1.08 ^ab^ (8)	10.28 ± 5.14 ^ab^ (66)	*H* = 16.04, *p* = 0.007
	Taxa count	4	3	3	5	5	5	
	Individuals	66	24	26	205	186	228	
	Shannon (^1^*D*)	1.18 (3.3)	0.96 (2.6)	0.73 (2.1)	1.21 (3.4)	1.01 (2.8)	1.30 (3.7)	
	Dominance (^2^*D*)	0.37 (2.7)	0.42 (2.4)	0.55 (1.8)	0.36 (2.8)	0.47 (2.1)	0.32 (3.1)	
	Evenness	0.82	0.87	0.69	0.67	0.55	0.74	
	Margalef	0.72	0.63	0.61	0.75	0.77	0.74	

* Note that the mean pollination count ± SE in the same row of a pollinator type followed by the same letters as superscripts are not significantly different (*p* > 0.05). The ^1^*D* and ^2^*D* within parentheses after the diversity indices represent first-order and second-order Hill numbers, which reflect the effective number of families based on the exponential of the Shannon diversity index and the effective number of dominant families by accounting for family abundance, respectively.

**Table 2:** The total number of pollinators captured from pan traps on the monocrop and intercrop treatments.

Family	Species	Treatments					
		Monocrops			Intercrops		
		Cowpea	Hemp	Watermelon	Hemp + Cowpea	Hemp + Watermelon	Watermelon + Cowpea
Cantharidae	*Chauliognathus pensylvanicus*	0	0	0	1	0	0
Syrphidae	*Toxomerus politus*	0	0	0	0	0	1
Andrenidae	*Calliopsis* species	0	0	1	0	0	0
Apidae	*Apis mellifera*	0	0	1	4	2	2
	*Bombus impatiens*	3	3	2	2	3	4
	*Bombus pensylvanicus*	0	3	1	0	1	0
	*Ceratina strenua*	1	0	0	0	2	1
	*Melissodes bimaculatus*	1	1	0	0	1	0
	*Ptilothrix bombiformis*	0	0	0	1	1	1
	*Svastra obliqua*	0	0	0	0	0	1
Crabronidae	*Cerceris bicornuta*	0	0	0	0	0	3
	*Cerceris* sp. 1	0	0	0	0	1	0
	*Cerceris* sp. 2	1	0	0	0	0	0
Formicidae	* Ant	0	0	0	0	1	0
	*Brachymyrmex patagonicus*	0	0	1	1	0	0
	*Camponotus festinatus*	0	0	0	1	0	0
	*Lasius neoniger*	0	0	0	0	0	3
	*Linepithema humile*	2	0	0	0	0	0
	*Monomorium* sp.	2	0	1	1	0	1
	*Pheidole* species	0	0	0	0	1	0
	*Plagiolepsis* species	0	0	0	0	0	1
	*Solenopsis* sp.	2	0	0	0	0	1
Halictidae	*Agapostemon sericeus*	0	0	1	4	5	1
	*Agapostemon virescens*	0	4	4	11	7	9
	*Augochlora pura*	3	4	2	7	6	12
	*Halictus confusus*	1	0	0	6	2	1
	*Halictus rubicundus*	3	1	7	9	2	3
	*Halictus* sp.	3	0	0	0	0	2
	*Lasioglossum* sp. 1	1	5	4	1	1	1
	*Lasioglossum* sp. 2	19	5	5	11	3	6
	*Lasioglossum* sp. 3	1	0	0	0	0	0
	*Lasioglossum viridatum*	7	9	10	21	18	29
	* Sweat bee	0	0	0	1	0	0
Megachilidae	*Megachile rotundata*	0	0	0	0	0	1
	*Osmia* sp.	1	0	0	0	0	0
Pompilidae	* Spider wasp	1	0	0	0	0	0
	*Anoplius americanus*	1	0	0	0	0	0
	*Anoplius moestus*	1	0	0	2	0	0
	*Anoplius* sp. 1	0	0	0	1	0	0
	*Anoplius* sp. 2	0	0	0	2	0	1
	*Aporinellus* sp.	0	0	1	2	0	1
Scoliidae	*Scolia dubia*	0	0	0	0	1	0
Sphecidae	*Chalybion* sp.	1	0	0	0	0	0
	*Sceliphron destillatorium*	0	0	0	0	1	0
Tiphiidae	*Tiphia* sp.	1	0	0	1	0	3
Vespidae	* Wasp_1_	0	0	0	0	0	1
	* Wasp_2_	1	0	0	0	0	0
	*Parancistrocerus fulvipes*	0	0	0	0	0	1
	*Polistes fuscatus*	0	0	0	0	0	1
	*Vespula squamosa*	0	0	2	1	1	2
Noctuidae	* Moth	3	0	3	1	0	2
	Taxa count	23	9	16	23	20	29
	Individuals	60	35	46	92	60	96
	Shannon (^1^*D*)	2.59 (13.3)	2.03 (7.6)	2.45 (11.6)	2.59 (13.3)	2.49 (12.1)	2.67 (14.4)
	Dominance (^2^*D*)	0.13 (7.7)	0.15 (6.7)	0.11 (9.1)	0.11 (9.1)	0.13 (7.7)	0.13 (7.7)
	Evenness	0.58	0.84	0.73	0.58	0.60	0.50
	Margalef	5.37	2.25	3.92	4.87	4.64	6.14

Note that the unidentified insects beyond family levels, owing to taxonomic impediments (absence of suitable identification materials), are represented with asterisks (*) and a common name. Also, the ^1^*D* and ^2^*D* within parentheses after the diversity indices represent first-order and second-order Hill numbers, which reflect the effective number of families based on the exponential of the Shannon diversity index and the effective number of dominant families by accounting for family abundance, respectively.

**Table 3:** The total number of pollinators captured from sticky cards on the monocrop and intercrop treatments.

Family	Treatments					
	Monocrops			Intercrops		
	Cowpea	Hemp	Watermelon	Hemp + Cowpea	Hemp + Watermelon	Watermelon + Cowpea
Muscidae	0	1	1	0	40	3
Sarcophagidae	290	46	133	370	164	367
Syrphidae	4	1	5	13	9	29
Calliphoridae	0	0	0	0	3	3
Apidae	3	0	0	0	2	3
Chrysididae	0	0	0	5	0	0
Crabronidae	7	0	5	7	5	8
Formicidae	0	0	1	19	8	17
Halictidae	68	35	47	78	88	110
Pompilidae	17	0	1	18	1	10
Sphecidae	3	1	0	11	1	12
Vespidae	26	13	18	23	21	40
Noctuidae	0	1	0	0	0	2
Nymphalidae	0	1	0	0	0	0
Gelechiidae	0	3	0	0	3	4
Pieridae	0	1	0	0	0	0
Taxa count	8	10	8	9	12	13
Individuals	418	103	211	544	345	608
Shannon (^1^*D*)	1.04 (2.9)	1.36 (3.9)	1.09 (3.0)	1.17 (3.2)	1.51 (4.5)	1.37 (3.9)
Dominance (^2^*D*)	0.51 (2.0)	0.33 (3.0)	0.46 (2.2)	0.49 (2.0)	0.31 (3.2)	0.41 (2.4)
Evenness	0.35	0.39	0.37	0.36	0.38	0.30
Margalef	1.16	1.94	1.31	1.27	1.88	1.87

Note that the ^1^*D* and ^2^*D* within parentheses after the diversity indices represent first-order and second-order Hill numbers, which reflect the effective number of families based on the exponential of the Shannon diversity index and the effective number of dominant families by accounting for family abundance, respectively.

**Table 4:** The total composition (class, order, and family) of arthropods, their functional traits, distribution, proportion, and diversity indices across six selected cropping systems and alongside the proportion of key groups (pollinators, herbivores, and predators) within the systems.

S/n	Class (%)	Order (%)	Family (%)	Functional Trait		Total Count (Proportion Across Systems) ^a^					
					Monocrop			Intercrop			
					Cowpea	Hemp	Watermelon	Hemp + Cowpea	**Hemp + Watermelon**	**Watermelon + Cowpea**	**Total**
**1**	Arachnida (0.07) ^2^	Araneae (0.07) ^8^	* Spider (60.9)	p	2 (14.29)	5 (35.71)	1 (7.14)	1 (7.14)	2 (14.29)	3 (21.43)	14
2			**Salticidae** (17.4)	p	2 (50)	0 (0)	1 (25)	0 (0)	1 (25)	0 (0)	4
3			Tetragnathidae (4.35)	p	0 (0)	0 (0)	0 (0)	0 (0)	1 (100)	0 (0)	1
4			**Thomisidae** (17.4)	p	0 (0)	0 (0)	1 (25)	3 (75)	0 (0)	0 (0)	4
5	Insecta (99.93) ^1^	Blattodea (0.003) ^11^	Kalotermitidae (100)	d	0 (0)	0 (0)	0 (0)	1 (100)	0 (0)	0 (0)	1
6		Coleoptera (6.8) ^5^	Bostrichidae (0.05)	h	0 (0)	1 (100)	0 (0)	0 (0)	0 (0)	0 (0)	1
7			Cantharidae (0.05)	po, p, h	0 (0)	0 (0)	0 (0)	1 (100)	0 (0)	0 (0)	1
8			Carabidae (6.22)	p, h	46 (34.07)	3 (2.22)	3 (2.22)	36 (26.67)	6 (4.44)	41 (30.37)	135
9			**Chrysomelidae** (45.49)	h	92 (9.31)	80 (8.10)	269 (27.23)	136 (13.77)	221 (22.37)	190 (19.23)	988
10			**Coccinellidae** (22.97)	p	84 (16.83)	20 (4.01)	72 (14.43)	90 (18.04)	91 (18.04)	143 (28.66)	499
11			Curculionidae (14.69)	h	61 (19.12)	31 (9.72)	80 (25.08)	53 (16.61)	35 (10.97)	59 (18.50)	319
12			Elateridae (1.75)	h, p	2 (5.26)	4 (10.53)	13 (34.21)	5 (13.16)	8 (21.05)	6 (15.79)	38
13			Hydrophilidae (3.18)	h, p	8 (11.59)	24 (34.78)	2 (2.90)	17 (24.64)	16 (23.19)	2 (2.90)	69
14			Lampyridae (0.97)	p	0 (0)	3 (14.29)	2 (9.52)	4 (19.05)	1 (4.76)	11 (52.38)	21
15			Mordellidae (2.81)	h, p	8 (13.12)	0 (0)	10 (16.39)	14 (22.95)	14 (22.95)	15 (24.59)	61
16			Scarabaeidae (1.11)	d, h	3 (12.50)	1 (4.17)	4 (16.67)	8 (33.33)	3 (12.50)	5 (20.83)	24
17			Staphylinidae (0.74)	p	7 (43.75)	1 (6.25)	0 (0)	4 (25.0)	2 (12.50)	2 (12.50)	16
18		Diptera (36.81) ^1^	Calliphoridae (0.05)	po	0 (0)	0 (0)	0 (0)	0 (0)	3 (50)	3 (50)	6
19			Ceratopogonidae (0.09)	pa, n, h	2 (18.18)	0 (0)	1 (9.09)	6 (54.55)	2 (18.18)	0 (0)	11
20			Chironomidae (0.02)	d	0 (0)	0 (0)	0 (0)	1 (50)	0 (0)	1 (50)	2
21			**Chloropidae** (25.23)	d, h	456 (15.45)	209 (7.08)	176 (5.96)	775 (26.26)	353 (11.96)	982 (33.28)	2951
22			**Dolichopodidae** (50.33)	p	448 (7.61)	670 (11.38)	745(12.66)	1109 (18.84)	1264 (21.47)	1651 (28.05)	5887
23			Drosophilidae (3.57)	d, s	118 (28.30)	51 (12.23)	140(33.57)	24 (5.76)	32 (7.67)	52 (12.47)	417
24			Muscidae (0.39)	po	0 (0)	1 (2.22)	1 (2.22)	0 (0)	40 (88.89)	3 (6.67)	45
25			Phoridae (6.75)	n, d, h	105 (13.29)	216 (27.34)	94 (11.90)	76 (9.86)	66 (8.35)	233 (29.50)	790
26			Sarcophagidae (12.2)	p, po	294 (20.60)	51 (3.57)	138 (9.67)	387 (27.12)	171 (11.90)	386 (27.05)	1427
27			Sciaridae (0.72)	d, f	19 (22.61)	6 (7.14)	6 (7.14)	23 (27.38)	15 (17.86)	15 (17.86)	84
28			Syrphidae (0.53)	po	4 (6.65)	1 (1.61)	5 (8.07)	13 (20.96)	9 (14.52)	30 (48.38)	62
29			Tephritidae (0.12)	h	4 (28.57)	0 (0)	6 (42.86)	2 (14.29)	1 (7.14)	1 (7.14)	14
30		Hemiptera (19.43) ^3^	Acanaloniidae (0.02)	h	0 (0)	0 (0)	0 (0)	0 (0)	1 (100)	0 (0)	1
31			Aleyrodidae (4.21)	h	78 (30)	8 (3.08)	13 (5)	69 (26.53)	35 (13.46)	57 (21.92)	260
32			Anthocoridae (5.59)	p	66 (19.13)	13 (3.77)	50 (14.49)	73 (21.16)	52 (15.07)	91 (26.38)	345
33			**Aphididae** (19.35)	h	94 (7.87)	148 (12.40)	215 (18.01)	170 (14.24)	333 (27.89)	234 (19.60)	1194
34			Berytidae (0.08)	p, h	1 (20)	0 (0)	3 (60)	0 (0)	0 (0)	1 (20)	5
35			Blissidae (0.02)	h	0 (0)	0 (0)	0 (0)	0 (0)	1 (100)	0 (0)	1
36			**Cicadellidae (56.82)**	h	767 (21.87)	435 (12.40)	456 (13.00)	740 (21.10)	369 (10.52)	740 (21.10)	3507
37			Coreidae (0.26)	h	4 (25)	2 (12.50)	0 (0)	5 (31.25)	2 (12.50)	3 (18.75)	16
38			Cydnidae (0.03)	h	0 (0)	0 (0)	0 (0)	0 (0)	0 (0)	2 (100)	2
39			Derbidae (1.04)	h	15 (23.44)	6 (9.38)	8 (12.50)	14 (21.88)	6 (9.38)	15 (23.44)	64
40			Geocoridae (1.39)	p	11 (12.79)	14 (16.28)	12 (13.95)	23 (26.74)	18 (20.93)	8 (9.30)	86
41			Lygaeidae (0.11)	p	0 (0)	0 (0)	1 (14.29)	0 (0)	3 (42.86)	3 (42.86)	7
42			Membracidae (3.78)	h	45 (19.31)	14 (6.01)	18 (7.73)	57 (24.46)	37 (15.88)	62 (26.61)	233
43			Miridae (6.12)	h, p	33 (8.73)	103 (27.25)	44 (11.64)	93 (24.60)	52 (13.76)	53 (14.02)	378
44			Pentatomidae (0.76)	h	5 (10.64)	3 (6.38)	1 (2.13)	8 (17.02)	11 (23.40)	19 (40.43)	47
45			Plataspidae (0.02)	h	0 (0)	0 (0)	0 (0)	0 (0)	1 (100)	0 (0)	1
46			Psyllidae (0.19)	h	1 (8.33)	4 (33.30)	0 (0)	2 (16.70)	0 (0)	5 (41.70)	12
47			Reduviidae (0.07)	p	0 (0)	0 (0)	0 (0)	1 (25)	1 (25)	2 (50)	4
48			Rhopalidae (0.07)	h	1 (25)	0 (0)	2 (50)	1 (25)	0 (0)	0 (0)	4
49			Thyreocoridae (0.05)	h	0 (0)	3 (100)	0 (0)	0 (0)	0 (0)	0 (0)	3
50			Tingidae (0.03)	h	0 (0)	0 (0)	1 (50)	0 (0)	0 (0)	1 (50)	2
51		Hymenoptera (11.58) ^4^	Andrenidae (0.03)	po	0 (0)	0 (0)	1 (100)	0 (0)	0 (0)	0 (0)	1
52			Apidae (13.7)	po	27 (5.35)	28 (5.56)	23 (4.56)	111 (22.02)	168 (33.30)	147 (29.17)	504
53			Braconidae (16.4)	p	100 (16.53)	36 (5.95)	72 (11.90)	160 (26.50)	113 (18.70)	124 (20.50)	605
54			Chalcididae (0.27)	p	0 (0)	0 (0)	0 (0)	5 (50)	2 (20)	3 (30)	10
55			Chrysididae (0.22)	p, po	1 (12.5)	1 (12.50)	0 (0)	5 (62.50)	0 (0)	1 (12.50)	8
56			Crabronidae (1.01)	p, po	8 (21.6)	0 (0)	5 (13.50)	7 (18.90)	6 (16.20)	11 (29.70)	37
57			Cynipidae (2.23)	h	19 (23.20)	20 (20.40)	5 (6.10)	10 (12.20)	13 (15.90)	15 (18.30)	82
58			Encyrtidae (0.95)	p	0 (0)	22 (62.90)	6 (17.10)	4 (11.40)	2 (5.70)	1 (2.90)	35
59			Eurytomidae (0.05)	p	0 (0)	2 (100)	0 (0)	0 (0)	0 (0)	0 (0)	2
60			Evaniidae (0.65)	p	2 (8.3)	1 (4.20)	5 (20.80)	4 (16.70)	5 (20.80)	7 (29.20)	24
61			Figitidae (0.73)	p	5 (18.50)	1 (3.7)	0 (0)	6 (22.20)	8 (29.60)	7 (25.90)	27
62			Formicidae (2.20)	p, po, d	12 (14.80)	0 (0)	4 (5)	25 (30.90)	12 (14.80)	28 (34.60)	81
63			**Halictidae** (20.90)	po	117 (15.20)	63 (8.20)	87 (11.30)	157 (20.40)	151 (19.60)	194 (25.20)	769
64			Ichneumonidae (0.11)	p	1 (25)	0 (0)	1 (25)	0 (0)	0 (0)	2 (50)	4
65			Megachilidae (0.05)	po	1(50)	0 (0)	0 (0)	0 (0)	0 (0)	1 (50)	2
66			Pompilidae (1.63)	p, po	20 (33.30)	0 (0)	2 (3.30)	25 (41.70)	1 (1.70)	12 (20)	60
67			Pteromalidae (0.95)	p	19 (54.30)	0 (0)	3 (8.60)	6 (17.10)	4 (11.40)	3 (8.60)	35
68			**Scelionidae** (26.8)	p	79 (7.80)	50 (5.10)	169 (17.10)	236 (23.90)	183 (18.50)	271 (27.40)	988
69			Scoliidae (0.03)	p, po	0 (0)	0 (0)	0 (0)	0 (0)	1 (100)	0 (0)	1
70			Sphecidae (0.84)	p, po	4 (12.90)	1 (3.20)	0 (0)	11 (35.50)	3 (9.70)	12 (38.70)	31
71			Tiphiidae (0.71)	p, po	9 (34.60)	2 (7.70)	2 (7.70)	4 (15.40)	3 (11.50)	6 (23.10)	26
72			Vespidae (9.46)	p, po	63 (18.10)	16 (4.60)	20 (5.80)	108 (31)	30 (8.60)	111 (31.90)	348
73		Lepidoptera (0.076) ^7^	**Gelechiidae** (41.67)	po, n	0 (0)	3 (30)	0 (0)	0 (0)	3 (30)	4 (40)	10
74			**Noctuidae** (50)	h, po	3 (25)	1 (8.3)	3 (25)	1 (8.30)	0 (0)	4 (33.40)	12
75			Nymphalidae (4.17)	po	0 (0)	1 (100)	0 (0)	0 (0)	0 (0)	0 (0)	1
76			Pieridae (4.17)	po	0 (0)	1 (100)	0 (0)	0 (0)	0 (0)	0 (0)	1
77		Odonata (0.0095) ^9^	* Dragonfly (33.33)	p	0 (0)	0 (0)	0 (0)	1 (100)	0 (0)	0 (0)	1
78			Libellulidae (66.67)	p	0 (0)	0 (0)	0 (0)	0 (0)	1 (50)	1 (50)	2
79		Orthoptera (0.0063) ^10^	**Acrididae** (50)	h	0 (0)	0 (0)	0 (0)	0 (0)	0 (0)	1 (100)	1
80			**Gryllidae** (50)	h	0 (0)	0 (0)	0 (0)	0 (0)	0 (0)	1 (100)	1
81		Psocodea (0.55) ^6^	**Philotarsidae** (100)	h, f	14 (8.10)	14 (8.10)	22 (12.70)	30 (17.30)	42 (24.30)	51 (29.50)	173
82		Thysanoptera (24.64) ^2^	**Thripidae** (100)	h	601 (7.70)	2035 (26)	881 (11.30)	1816 (24)	1278 (16.30)	1217 (15.60)	7828
			Taxa count		53	50	53	58	60	64	**Total**
			Individuals		3991	4429	3905	6777	5307	7365	**31,774**
			Pollinator ᶧ (%)		446 (11.2)	104 (2.4)	204 (5.2)	698 (10.3)	447 (8.4)	755 (10.2)	
			Herbivore ᶧ (%)		2468 (61.8)	3365 (75.9)	2330 (59.7)	4145 (61.2)	2906 (54.6)	4015 (54.5)	
			Predator ᶧ (%)		1337 (33.5)	1043 (23.5)	1388 (35.5)	2474 (36.5)	2078 (39.2)	3018 (41.0)	
			Others ᶧ (%)		727 (18.2)	497 (11.2)	446 (11.4)	963 (14.2)	523 (9.9)	1367 (18.6)	
			Shannon (^1^*D*)		2.79 (16.3)	2.06 (7.8)	2.60 (13.5)	2.57 (13.0)	2.56 (13.0)	2.65 (14.2)	
			Dominance (^2^*D*)		0.10 (10.0)	0.25 (4.0)	0.12 (8.3)	0.13 (7.7)	0.14 (7.1)	0.12 (8.3)	
			Evenness		0.31	0.16	0.26	0.23	0.22	0.22	
			Margalef		6.27	5.84	6.29	6.46	6.88	7.08	

Note that the numeric superscripts following specific class and order represent an order of abundance within such taxon relative to the total of sampled individuals, while the family is relative to the total individuals within an order, where superscript ^1^ presumes a greater proportion than superscript ^2^, likewise ^3–11^ and ^11^ is the least proportion. * Unable to identify to family; The letter codes: d = detritivore, f = fungivore, h = herbivore, n = nectivore p = predator, po = pollinator, s = saprophage. ᶧ indicates that the percentages were within each system and may contain overlapping functional traits. Families in bold print represents the first two major taxa within an order. The ^1^*D* and ^2^*D* within parentheses after the diversity indices represent first-order and second-order Hill numbers, which reflect the effective number of families based on the exponential of the Shannon diversity index and the effective number of dominant families by accounting for family abundance, respectively. Superscript ^a^ indicates that these are aggregates of all three sampling techniques.

## Data Availability

The original contributions presented in this study are included in the article. Further inquiries can be directed to the corresponding author.

## References

[1] Gebru H. (2015). A review on the comparative advantages of intercropping to mono-cropping system. J. Biol. Agric. Healthc..

[2] Miller G., Greene J. (2018). Intercropping seedless watermelon and cotton. Hortscience.

[3] Ju Q., Ouyang F., Gu S., Qiao F., Yang Q., Qu M., Ge F. (2019). Strip intercropping peanut with maize for peanut aphid biological control and yield enhancement. Agric. Ecosyst. Environ..

[4] Ouyang F., Su W., Zhang Y., Liu X., Su J., Zhang Q., Men X., Ju Q., Ge F. (2020). Ecological control service of the predatory natural enemy and its maintaining mechanism in rotation-intercropping ecosystem via wheat-maize-cotton. Agric. Ecosyst. Environ..

[5] Glaze-Corcoran S., Hashemi M., Sadeghpour A., Jahanzad E., Afshar R.K., Liu X., Herbert S.J. (2020). Understanding intercropping to improve agricultural resiliency and environmental sustainability. Adv. Agron..

[6] Dingha B.N., Jackai L.E., Amoah B.A., Akotsen-Mensah C. (2021). Pollinators on cowpea *Vigna unguiculata*: Implications for intercrop ping to enhance biodiversity. Insects.

[7] Dingha B.N., Omaliko P.C., Amoah B.A., Jackai L.E., Shrestha D. (2021). Evaluation of cowpea (*Vigna unguiculata*) in an intercropping system as pollinator enhancer for increased crop yield. Sustainability.

[8] Dingha B.N., Mukoko G.N., Egbon I.N., Jackai L.E. (2024). Intercropping industrial hemp and cowpea enhances the yield of squash -a pollinator dependent crop. Agriculture.

[9] Katumo D.M., Liang H., Ochola A.C., Lv M., Wang Q., Yang C. (2022). Pollinator diversity benefits natural and agricultural ecosystems, environmental health, and human welfare. Plant Divers..

[10] Corlett R.T. (2020). Safeguarding our future by protecting biodiversity. Plant Divers..

[11] Ouyang F., Men X., Yang B., Su J., Zhang Y., Zhao Z., Ge F. (2012). Maize benefits the predatory beetle, *Propylea japonica* (Thunberg), to provide potential to enhance biological control for aphids in cotton. PLoS ONE.

[12] Yang Q., Men X., Zhao W., Li C., Zhang Q., Cai Z., Ge F., Ouyang F. (2021). Flower strips as a bridge habitat facilitate the movement of predatory beetles from wheat to maize crops. Pest Manag. Sci..

[13] Dingha B.N., Jackai L.E. (2023). Chemical composition of four industrial hemp (*Cannabis sativa* L.) pollen and bee preference. Insects.

[14] Dingha B.N., Jackai L.E.N. (2025). The potential impact of flower characteristics and pollen viability of four industrial hemp (*Cannabis sativa* L.) grain varieties on cross-pollination. Agronomy.

[15] Bland R.G., Jaques H.E. (1978). How to Know the Insects.

[16] Hölldobler B., Wilson E.O. (1990). The Ants.

[17] Hammer O. (2016). PAST: PAleontological Statistics, v. 3.13 Reference Manual.

[18] Chao A., Gotelli N.J., Hsieh T.C., Sander E.L., Ma K.H., Colwell R.K., Ellison A.M. (2014). Rarefaction and extrapolation with Hill numbers: A framework for sampling and estimation in species diversity studies. Ecol. Monogr..

[19] Shannon C.E. (1948). A mathematical theory of communication. Bell Syst. Tech. J..

[20] Magurran A.E. (2004). Measuring Biological Diversity.

[21] de Mendiburu F. (2023). agricolae: Statistical Procedures for Agricultural Research. R Package Version 1.3-7. https://CRAN.R-project.org/package=agricolae.

[22] Brooks M.E., Kristensen K., van Benthem K.J., Magnusson A., Berg C.W., Nielsen A., Skaug H.J., Maechler M., Bolker B.M. (2017). glmmTMB balances speed and flexibility among packages for zero-inflated generalized linear mixed modeling. R J..

[23] Lenth R. (2024). emmeans: Estimated Marginal Means, aka Least-Squares Means. R Package Version 1.10.4. https://CRAN.R-project.org/package=emmeans.

[24] Hothorn T., Bretz F., Westfall P. (2008). Simultaneous inference in general parametric models. Biom. J..

[25] Ogle D.H., Doll J.C., Wheeler A.P., Dinno A. (2023). FSA: Simple Fisheries Stock Assessment Methods. R Package Version 0.9.5. https://CRAN.R-project.org/package=FSA.

[26] R Core Team (2024). R: A Language and Environment for Statistical Computing.

[27] Slowikowski K. (2024). ggrepel: Automatically Position Non-Overlapping Text Labels with ‘ggplot2’. R Package Version 0.9.6. https://CRAN.R-project.org/package=ggrepel.

[28] Igbinosa B.I., Oigiangbe N.O., Egbon N.I. (2007). Insect pests of rain-fed upland rice and their natural enemies in Ekpoma, Edo State, Nigeria. Int. J. Trop. Insect Sci..

[29] Ajayi O.S., Samuel-Foo M. (2021). Hemp pest spectrum and potential relationship between *Helicoverpa zea* infestation and hemp production in the United States in the face of climate change. Insects.

[30] Buss E.A., Skelley P.E. (2023). An Initial List of Arthropods on Hemp (Cannabis sativa L.; Cannabaceae) in Florida.

[31] Ahmed M.Z., McKenzie1 C.L., Osborne L.S. (2024). Arthropod and mollusk pests of hemp, *Cannabis sativa* (Rosales: Cannabaceae), and their indoor management plan in Florida. J. Integr. Pest Manag..

[32] O’Brien C., Arathi H.S. (2019). Bee diversity and abundance on flowers of industrial hemp (*Cannabis sativa* L.). Biomass Bioenergy.

[33] Henne C.S., Rodriguez E., Adamczyk J.J. (2012). A survey of bee species found pollinating watermelons in the Lower Rio Grande Valley of Texas. Psyche.

[34] Campbell J.W., Stanley-Stahra C., Bammera M., Daniels J.C., Ellis J.D. (2019). Contribution of bees and other pollinators to watermelon (*Citrullus lanatus* Thunb.) pollination. J. Apic. Res..

[35] Huey S., Nieh J.C. (2017). Foraging at a safe distance: Crab spider effects on pollinators. Ecol. Entomol..

[36] Pekár S., Šoltysová V., Booysen R., Arnedo M. (2025). Evolution of spider- and ant-eating habits in crab spiders (Araneae: Thomisidae). Zool. J. Linn. Soc..

[37] Gavini S.S., Quintero C. (2024). Predation risk and floral rewards: How pollinators balance these conflicts and the consequences on plant fitness. Curr. Res. Insect Sci..

[38] Garantonakis N., Varikou K., Birouraki A., Edwards M., Kalliakaki V., Andrinopoulos F. (2016). Comparing the pollination services of honey bees and wild bees in a water melon field. Sci. Hortic..

[39] Adlerz W.C. (1966). Honey bee visit numbers and water melon pollination. J. Econ. Entomol..

[40] Moradeshaghi M.J., Bohart G.E. (1968). The biology of *Euphytomima nomiivora* (Diptera: Sarcophagidae), a parasite of the Alkali Bee, *Nomia melanderi* (Hymenoptera: Halictidae). J. Kansas Entomol. Soc..

[41] Young O.P., Edward G.B. (1990). Spiders in united states field crops and their potential effect on crop pests. J. Arachnol..

[42] Peterson J.A., Romero S.A., Harwood J.D. (2010). Pollen interception by linyphiid spiders in a corn agroecosystem: Implications for dietary diversification and risk-assessment. Arthropod-Plant Interact..

[43] Ulrich H. (2004). Predation by adult Dolichopodidae (Diptera): A review of literature with an annotated prey-predator list. Studia Dipterol..

[44] Cicero J.M., Adair M.M., Adair R.C., Hunter W.B., Avery P.B., Mizell R.F. (2017). Predatory behavior of long-legged flies (Diptera: Dolichopodidae) and their potential negative effects on the parasitoid biological control agent of the Asian citrus psyllid (Hemiptera: Liviidae). Fla. Entomol..

[45] Arnold S.E.J., Elisante F., Mkenda P.A., Tembo Y.L.B., Ndakidemi P.A., Gurr G.M., Darbyshire I.A., Belmain S.R., Stevenson P.C. (2021). Beneficial insects are associated with botanically rich margins with trees on small farms. Sci. Rep..

[46] Loughridge A.H., Luff M.L. (1983). Aphid predation by *Herpalus rufipes* (Degeer) (Coleoptera: Carabidae) in the laboratory and field. J. Appl. Ecol..

[47] Quellhorst H., Athanassiou C.G., Zhu K.Y., Morrison W.R. (2021). The biology, ecology and management of the larger grain borer, Prostephanus truncatus (Horn) (Coleoptera: Bostrichidae). J. Stored Prod. Res..

[48] Losey J.E., Vaughan M. (2006). The economic value of ecological services provided by insects. BioScience.

[49] Mdellel L., Zouari S., Jouini J.G., Adouani R., Halima M.K.B. (2024). Checklist and distribution of coccinellid aphid predators in Tunisia. Jordan J. Nat. Hist..

[50] United States Sustainability Alliance (USSA) (2024). U.S. Sustainable Agriculture: Laws, Policies, and Programs.

[51] Ajayi E.O., Adeoye I.B., Shittu O.A. (2017). Economic analysis of intercropping okra with legumes. J. Agric. Sci..

[52] Legodi K.D., Ogola J.B.O. (2020). Cassava-legume intercrop: I. Effects of relative planting dates of legumes on cassava productivity. Acta Agric. Scand. Sect. B Soil Plant Sci..

[53] Fedeli S.B., Leibler S. (2024). Toward systems agroecology: Design and control of intercropping. Proc. Natl. Acad. Sci. USA.

[54] Ogola J.B.O., Mathews C., Magongwa S.M. (2013). The productivity of cassava-legume intercropping system in a dry environment in Nelspruit, South Africa. Afr. Crop Sci. Conf. Proc..

[55] Taah K.J., Buah J.N., Ogyiri A.E. (2017). Evaluation of spatial arrangement of legumes on weed suppression in cassava production. J. Agric. Biol. Sci..

[56] Landis D.A., Gardiner M.M., Van Der Werf W., Swinton S.M. (2008). Increasing corn for biofuel production reduces biocontrol services in agricultural landscapes. Proc. Natl. Acad. Sci. USA.

[57] Mattias J., Riccardo B., Barbara E., Henrik G.S., Jan B., Berta C.L., Camilla W., Ola O. (2014). Ecological production functions for biological control services in agricultural landscapes. Methods Ecol. Evol..

[58] Blackwell B.F., Klug P.E., Humberg L.A., Brym Z.T., Kluever B.M., Edwards J. (2022). Cultivation of industrial hemp on and near airports: Implications for wildlife use and risk to aviation safety. Hum.-Wildl. Interact..

